# Research on the Prediction of Green Plum Acidity Based on Improved XGBoost

**DOI:** 10.3390/s21030930

**Published:** 2021-01-30

**Authors:** Yang Liu, Honghong Wang, Yeqi Fei, Ying Liu, Luxiang Shen, Zilong Zhuang, Xiao Zhang

**Affiliations:** College of Mechanical and Electronic Engineering, Nanjing Forestry University, Nanjing 210037, China; lyang_03@njfu.edu.cn (Y.L.); hhw@njfu.edu.cn (H.W.); feiyeqi@njfu.edu.cn (Y.F.); shenluxiang@njfu.edu.cn (L.S.); zzl0702@njfu.edu.cn (Z.Z.); zx_xhx@njfu.edu.cn (X.Z.)

**Keywords:** green plum, pH, hyperspectral, prediction, supervised learning

## Abstract

The acidity of green plum has an important influence on the fruit’s deep processing. Traditional physical and chemical analysis methods for green plum acidity detection are destructive, time-consuming, and unable to achieve online detection. In response, a rapid and non-destructive detection method based on hyperspectral imaging technology was studied in this paper. Research on prediction performance comparisons between supervised learning methods and unsupervised learning methods is currently popular. To further improve the accuracy of component prediction, a new hyperspectral imaging system was developed, and the kernel principle component analysis—linear discriminant analysis—extreme gradient boosting algorithm (KPCA-LDA-XGB) model was proposed to predict the acidity of green plum. The KPCA-LDA-XGB model is a supervised learning model combined with the extreme gradient boosting algorithm (XGBoost), kernel principal component analysis (KPCA), and linear discriminant analysis (LDA). The experimental results proved that the KPCA-LDA-XGB model offers good acidity predictions for green plum, with a correlation coefficient (R) of 0.829 and a root mean squared error (RMSE) of 0.107 for the prediction set. Compared with the basic XGBoost model, the KPCA-LDA-XGB model showed a 79.4% increase in R and a 31.2% decrease in RMSE. The use of linear, radial basis function (RBF), and polynomial (Poly) kernel functions were also compared and analyzed in this paper to further optimize the KPCA-LDA-XGB model.

## 1. Introduction

Green plum contains a variety of natural acids, such as citric acid, which is indispensable for human metabolism. Green plum is a rare alkaline fruit that contains threonine and other amino acids and flavonoids, which are extremely beneficial to the normal progress of protein composition and metabolic functions for the human body and have obvious preventive and curative effects on widespread cardiovascular, urinary, and digestive diseases.

In actual production, the composition control of raw plums is mainly based on the experiences of workers by controlling the picking time. Generally speaking, the plums for producing plum essence are picked at a 70% ripe stage, while the plums for green plum wine are picked at an 80% ripe stage. However, due to the influence of region, variety, light, horticultural management, individual differences in the maturity of different plants, and the different parts of the fruit, there are still large differences in the total acid content of plums picked in the same batch. When measuring the acidity of green plums by physical and chemical tests, the electrode potential method is often used. However, this method is destructive, random, subjective, and has low detection efficiency, meaning that this method cannot meet the requirements for the detection and classification of raw fruits. Therefore, this article uses green plum as its research object to study a rapid, non-destructive detection method for the internal acidity of green plum based on hyperspectral imaging technology.

With the increasing research into spectral imaging technology in the field of agricultural and forestry product detection [1,2], detection objects and detection indicators are becoming increasingly more diversified [3,4]. For research on the internal quality detection of forest fruits, near-infrared spectroscopy, hyperspectral images [5], and multispectral images have been used in recent years to detect internal physical characteristics (e.g., hardness and sugar content) and engage in qualitative and quantitative analyses of chemical characteristics (e.g., acidity and volatile base nitrogen) [6]. Near-infrared spectroscopy technology is very widely used and can quickly and non-destructively predict the internal quality parameters of the fruit [7]. Wei [8] analyzed spectral data under a wavelength of 400~1000 nm to achieve the discrimination of persimmon maturity. The accuracy achieved using the linear model was 95.3%. Based on a combination of near-infrared spectroscopy technology and chemometric analysis methods, Ciccoritti [9] used partial least square regression (PLSR) [10,11] to predict the soluble solid content, dry matter, and titratable acidity of kiwifruit. The model determination coefficient $R^{2}$ values were 0.993, 0.983, and 0.933, respectively, and the model’s root mean square errors (RMSEs) were 0.40, 0.33, and 6.65, respectively, meaning that the prediction performance of the proposed model was good. However, the original spectral data of the tested sample have high dimensionality. When the original data are simultaneously input to the model for training, the complexity of the model will increase, and the efficiency of the model will decrease. At the same time, the high-dimensional information contained in the original spectrum may have a linear correlation between different dimensions, and redundant information may exist. When the full spectrum is used for modeling, the model’s prediction accuracy will decrease, and over-fitting will occur. Therefore, the feature band should be extracted before establishing a prediction model. Huang [12] proposed a method for detecting the pH of tomato based on the wavelength ratio and near-infrared spectroscopy. By using visible/short-wave near-infrared spectroscopy to establish a partial least squares (PLS) model that can evaluate tomato pH, the results showed that the PLS model offers good prediction ability, with a predicted correlation coefficient of 0.796. Shen [13] proposed a model combining sparse autoencoder (SAE) and partial least square regression (PLSR) (SAE-PLSR) to predict the soluble solid content of green plum. The correlation coefficient and the root mean square error of the prediction set were 0.9254 and 0.6827, respectively, which indicates excellent prediction performance.

With the development of machine learning technology, increasingly more machine learning models are being applied to spectral data processing. As a new research trend in machine learning, ensemble learning [14] integrates the results of multiple learning methods to improve the generalization ability and prediction accuracy of the original method. Research using estimation models represented by random forest (RF) [15] is also increasing. Li [16] used random forests to predict the sugar content of different types of fruits (e.g., apples and pears) and compared the prediction effects of PLS. The predicted $R^{2}$ increased from 0.731 of PLS to 0.888, and the root mean squared error in prediction set (RMSEP) reduced from 1.148 to 0.334. The results showed that random forest had obvious advantages in predicting the sugar content of various fruits. However, the use of gradient boosting algorithms such as gradient-boosted regression tree (GBRT) and XGBoost (extreme Gradient Boosting) has been rarely reported.

This study took the acidity of green plum as the research object. The purpose of the study is to predict the acidity of green plum through spectroscopy technology and machine learning algorithms, solve the current problem of relying on traditional destructive methods to detect the acidity of green plum, and realize the non-destructive detection of the acidity of green plum. To ensure the authenticity and reliability of hyperspectral data, a new hyperspectral imaging system based on the hyperspectral camera was developed. Using machine learning, the novel kernel principle component analysis—linear discriminant analysis—extreme gradient boosting algorithm (KPCA-LDA-XGB) model structure was proposed. In our KPCA-LDA-XGB model, the extreme gradient boosting algorithm in supervised learning was improved, and kernel principal component analysis (KPCA) and linear discriminant analysis (LDA) were used to extract the characteristics of the characteristic spectral curve of green plum and reduce the data dimension. XGBoost was also integrated to predict the green plum’s pH. The pH prediction performance of the proposed model was compared with that of the KPCA-XGB model, LDA-XGB model, and the improved kernel function model. The prediction results of the different models were visualized to reflect the pH predictions for each green plum, which could be conducive to the selection of high-quality green plum.

## 2. Experiments

### 2.1. Green Plum Samples

The green plum samples for the test were selected from Dali, Yunnan Province, China. In order to reflect the differences between samples, we purchased in small batches every week and delivered them by express on the day of picking. Due to the collision between the green plums during transportation and the defects of the green plums, the remaining 366 green plums were taken as examples after removing the green plums that had large areas of bad spots, rot, or were extremely small. The green plum samples were stored in a laboratory refrigerator and kept fresh at 4 °C to ensure refrigeration at a constant temperature. The samples for each test were randomly selected and placed in advance at room temperature. When the temperature of plums was the same as room temperature, spectral data collection and physical and chemical tests were performed using the plum samples.

### 2.2. Equipment

**Figure 1:**
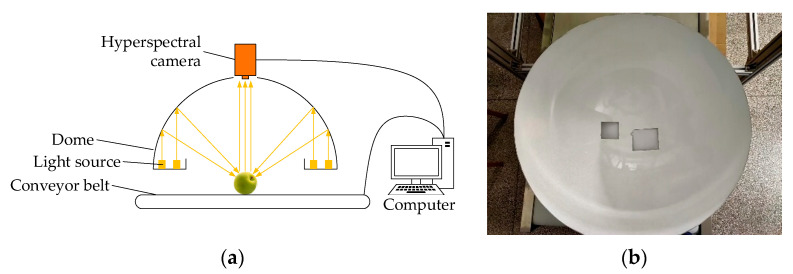
Hyperspectral imaging system and dome. (**a**) Hyperspectral imaging system. (**b**) Dome.

In order to obtain the hyperspectral data of green plums, a new green plum hyperspectral imaging system was developed, which was mainly composed of a hyperspectral camera (GaiaField-V10E-AZ4 camera, Sichuan Dualix Spectral Image Technology Co., Ltd., Chengdu, China), a light source, a conveyor belt, and a computer, as shown in Figure 1a. The parameters of the Gaiafield-V10E-AZ4 hyperspectral camera are shown in Table 1. The function of the conveyor belt in the system was to smoothly transport the green plum through the field of view of the camera. The conveyor belt was powered by a servo motor. Combined with the control of the computer, the speed of the conveyor belt could be matched with the camera’s shooting speed, so that the obtained data could be closer to reality. The interior of the dome was evenly coated with Teflon, which could diffuse the light emitted by the halogen light source for many times, so that the sample inside the dome was uniformly illuminated, as shown in Figure 1b. The light source system composed of the dome and the halogen lamp could meet the needs of the spectral range, and at the same time provide uniform and stable illumination to the sample, which was beneficial to eliminate the test errors caused by different illuminance conditions. The pH was measured using a pH meter (PHS-3E, Shanghai INESA Scientific Instrument Co., Ltd., China). The pH meter was ready for measurement after following the detailed calibration procedure described in the manual of the pH meter with pH buffers (pH = 4.00, pH = 6.86, Shanghai LICHEN-BX Instrument Technology Co., Ltd., China).

### 2.3. Hyperspectral Data Acquisition

Before each test, it was necessary to warm up the green plum hyperspectral imaging system and set the parameters of the system to the best. The tested green plum samples were placed on the conveyor belt for data collection when the system environment was stable. At the same time, the dark field spectral image of the system (*A_D_*) and spectral image of the standard reflectance calibration board with 99% reflectance (*A_W_*) were collected, and the reflectance calibration of the green plum spectral image was used to eliminate the unevenness of the light source and the dark noise of the camera. The calibration image *A_0_* is defined as:

(1) $$A_{0}=\frac{A-A_{D}}{A_{W}-A_{D}}\times 100\%$$

where *A* is the green plum spectral raw data to be corrected.

### 2.4. Green Plum pH Testing

The measurement of green plum acidity required squeezing out enough green plum juice. There was not much green plum juice that could be squeezed out from fresh green plums, so it was necessary to squeeze the green plum juice out of the green plum as soon as the spectrum data were collected to avoid the influence of subsequent physical and chemical tests of acidity due to the evaporation of water. The pH meter was cleaned with distilled water. The electrode was then inserted into the sample to test and record the pH value of each sample for subsequent data processing.

In total, 366 green plum samples were selected and sorted according to their pH values, and each of 4 samples in sequence are taken as a group. Within each group, random sampling was conducted at a ratio of 3:1 to obtain 274 calibration set samples, which were used to train the model and adjust model parameters, then 92 prediction set samples were used to evaluate the predictive ability of the model. Table 2 shows the pH distributions for the green plum.

### 2.5. Image Processing

**Figure 2:**
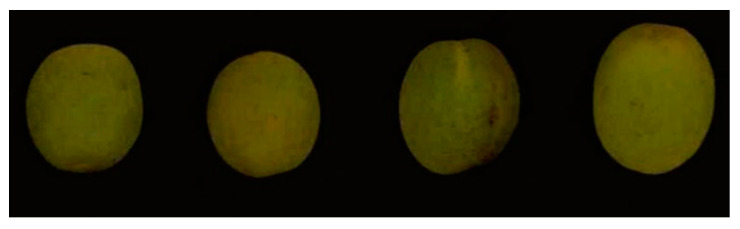
Pseudocolor images of some samples.

**Figure 3:**
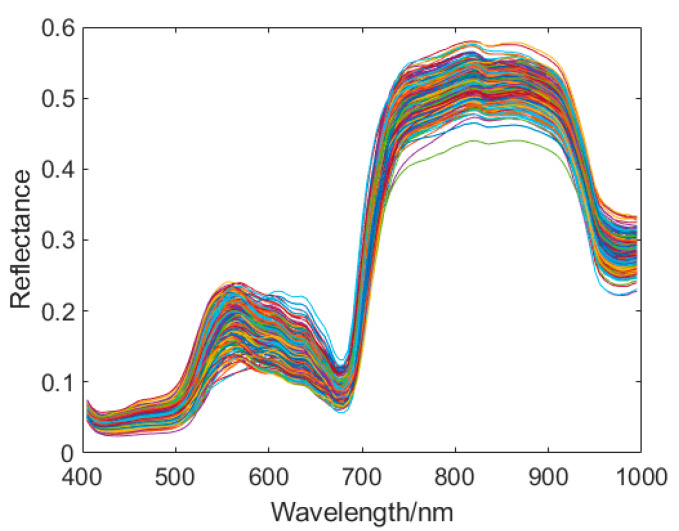
Original spectral reflectance curves of the plum samples.

A hyperspectral imaging system was used to collect the green plum images. The pseudocolor images were synthesized from the RGB spectrum. Figure 2 shows the pseudocolor images of the part of the samples. The ENVI5.3 software was used to calculate the average spectral reflectance of each band. The preprocessing, modeling, and analysis of green plum spectral data were realized by using JetBrains PyCharm 2019 software. All the codes were written in Python, using the deep learning framework Tensorflow to define the network calculation graph. Figure 3 shows the original spectral reflectance curves of all the green plum samples. The software, hardware, and compilation environment configuration of this experiment is shown in Table 3.

## 3. Model Establishment

### 3.1. XGBoost

The basic idea of ensemble learning is to combine multiple models rather than using a single model to solve a problem. Ensemble learning uses several different models as base classifiers and then combines those models to obtain better performance than using only one specific method. Due to the diversity of methods and the mutual restraint of errors, ensemble learning results provide better accuracy and robustness. Because of their advantages in improving models and their accuracy, ensemble learning algorithms have become an important research direction in the field of machine learning.

Bagging and Boosting are the most classic ensemble learning methods. The purpose of integrating base classifiers is to reduce overfitting and improve accuracy. The extreme gradient boosting algorithm (XGBoost) is a supervised gradient boosting-based ensemble learning algorithm proposed by Chen [17]. The goal of this algorithm is to create a K regression tree to obtain the predicted value of the tree group as close to the true value as possible and achieve the greatest generalization ability. The XGBoost algorithm is a kind of lifting algorithm that generates multiple weak learners through residual fitting. The generated weak classifiers are accumulated to obtain a strong learner [18,19]. XGBoost expands the loss function to the second order Taylor in the optimization process and introduces the second derivative information to make the model converge faster during the training process. In addition, XGBoost also adds a regularization term to the loss function to suppress model complexity and prevent overfitting. The specific derivation process of the XGBoost algorithm is as follows. Set $D=\left\{\left(x_{i},y_{i}\right)\right\}$ as a data set of n numbers of samples, where there are d numbers of features for each sample. $x_{i}$ represents the label of sample i. As a base model, we used a classification and regression tree (CART). The ensemble model of XGBoost uses an addition expression composed of K numbers of base models to predict the final result:

(2) $${\hat{y}}_{i}=\overset{K}{\underset{k=1}{\sum }}f_{k}\left(x_{i}\right)$$

where k is the number of the tree and$\;f_{k}\left(\cdot \right)$ is the expression of tree k.

The loss function can reflect the deviation of the model. In order to improve the generalization ability of the model, the model complexity was added to the optimization goal as a regularization term. The regularization term and the loss function of the model comprise the objective function of the XGBoost algorithm as follows:

(3) $$\{\begin{array}{l} Obj^{\left(k\right)}=\sum_{i=1}^{n}l\left[y_{i},{\hat{y}}_{i}^{\left(k-1\right)}+f_{t}\left(x_{i}\right)\right]+\sum_{k}\Omega \left(f_{k}\right)\\ \Omega \left(f\right)=\gamma T+\frac{1}{2}\lambda \|w{\|}^{2} \end{array}$$

where ${\hat{y}}_{i}^{\left(k-1\right)}$ is the sum of the output values of the previous (k−1) trees; $y_{i}$ is the real value;$\;{\hat{y}}_{i}$ is the predicted value; $f_{k}\left(x_{i}\right)$ is the output result of tree k; l is a differentiable convex loss function; Ω(·) is the penalty term; γ and λ are the regularization parameters of leaf weight and number, respectively; and w and T are the value and number of the leaf node, respectively. $I_{j}$ is defined as the sample set on leaf node j. The Taylor formula is used to expand the loss function at ${\hat{y}}_{i}^{\left(k-1\right)}$. $g_{i}$ and $h_{i}$ are the first and second derivatives of the Taylor expansion. After removing the constant term, the objective function after Taylor expansion becomes the following:

(4) $$\mathrm{Ob}j^{\left(k\right)}=\overset{T}{\underset{j=1}{\sum }}\left[\left(\sum_{i\in I_{j}}g_{i}\right)\omega_{j}+\frac{1}{2}\left(\sum_{i\in I_{j}}h_{i}+\lambda \right)\omega_{j}^{2}\right]+\gamma T$$

where $\omega_{j}$ is the weight of leaf node j. Next, we set $G_{i}=\underset{i\in I_{j}}{\sum }g_{i},\;\;H_{i}=\underset{i\in I_{j}}{\sum }h_{i}$ and substitute them into equation (3). The objective function is then simplified as:

(5) $$\mathrm{Ob}j^{\left(k\right)}=\overset{T}{\underset{j=1}{\sum }}\left[G_{i}\omega_{j}+\frac{1}{2}\left(H_{i}+\lambda \right)\omega_{j}^{2}\right]+\gamma T$$

In Equation (4), the leaf node $\omega_{j}$ is an uncertain value. Therefore, the objective function $\mathrm{Ob}j^{\left(k\right)}$ is calculated for the first derivative of $\omega_{j}$, and the optimal value $\omega_{j}^{*}$ of the leaf node j is solved as:

(6) $$\omega_{j}^{*}=-\frac{G_{i}}{H_{i}+\lambda }$$

After substituting $\omega_{j}^{*}$ back into the objective function, the minimum value of $\mathrm{Ob}j^{\left(k\right)}$ is obtained as:

(7) $$\mathrm{Ob}j^{\left(k\right)}=-\frac{1}{2}\overset{T}{\underset{j=1}{\sum }}\frac{G_{j}^{2}}{H_{i}+\lambda }+\;\gamma T$$

When building CART, XGBoost uses a greedy algorithm to split features. We next set the maximum depth of the tree to m and the algorithm flow as shown in Algorithm 1.
**Algorithm 1:Exact Greedy Algorithm for Split Finding.**Input: I, instance set of the current nodeInput: d, feature dimension gain←0$G\leftarrow \underset{i\in I}{\sum }g_{i},H\leftarrow \underset{i\in I}{\sum }h_{i}$for k=1 to m do $G_{L}\leftarrow 0,H_{L}\leftarrow 0$for $j\;in\;sorted\left(I,by\;x_{jk}\right)$ do                                                $G_{L}\leftarrow G_{L}+g_{j},H_{L}\leftarrow H_{L}+h_{j}$
                                               $G_{R}\leftarrow G-G_{L},H_{R}\leftarrow H-H_{L}$
                                 $score\leftarrow max\left(score,\frac{G_{L}^{2}}{H_{L}+\lambda }+\frac{G_{R}^{2}}{H_{R}+\lambda }-\frac{G^{2}}{H+\lambda }\right)$endendOutput: Split with max score

**Figure 4:**
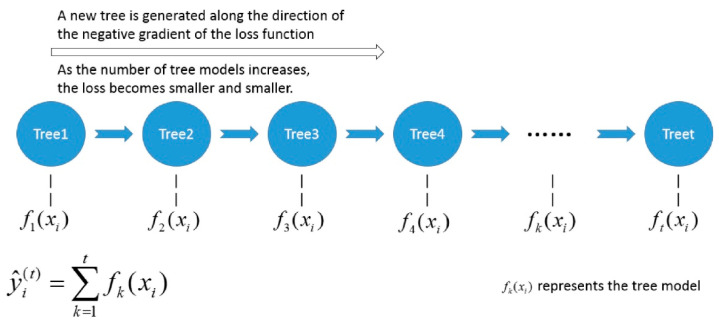
A schematic diagram of the XGBoost algorithm.

During each iteration, the greedy algorithm traverses all the features of each node from the root node. The point with the highest score is selected as the split node. Splitting stops after splitting to the maximum depth of the tree and starting to build the residua of the next tree. Finally, all the generated trees are gathered to obtain the XGBoost model. The schematic diagram of the construction of the XGBoost algorithm is shown in Figure 4 [20].

XGBoost improves the Gradient Boosting Decision Tree (GBDT) in three main ways. First, the residual used in traditional GBDT is the result of using the first-order Taylor expansion, while XGBoost uses the second-order Taylor expansion with the first and second orders as improved residuals. Consequently, the XGBoost model has more extensive applications. Secondly, XGBoost adds a regularization term to the objective function to control the complexity of the model. This regularization term can reduce the variance and overfitting of the model. Finally, XGBoost also references the practice of the random forest column sampling method to further reduce overfitting. XGBoost has shown excellent learning performance with efficient training speed.

### 3.2. KPCA-LDA-XGB

Linear discriminant analysis (LDA), also known as Fisher discriminant analysis, is a type of supervised learning classification and dimensionality reduction method. LDA can effectively use the categorical information of the original data to perform accurate feature extraction. By seeking projection transformation, LDA achieves a high degree of differentiation between classes and a high degree of similarity within classes, making it more efficient for feature extraction.

Kernel Principle Component Analysis (KPCA) is a non-linear feature extraction method. The process of non-linear mapping is completed in KPCA through the kernel function. The dimensionality reduction of non-linear data is achieved while retaining the original data information to the greatest extent. KPCA is more effective for capturing the non-linear characteristics of the data.

Considering the problem of green plum pH prediction, to further improve the feature extraction ability of our model, KPCA and LDA were used to reduce the dimensionality of the input of XGBoost. In KPCA, we first used nonlinear mapping Փ:R→F to map the data in the original input space to the high-dimensional feature space F. Then, principal component analysis was performed on the data in feature space F [21]. The kernel principal with a relatively large cumulative contribution rate was next selected to form a new data set to reduce the dimensionality of the data while retaining the nonlinear information between variables. Since the specific form of the mapping function Փ was generally unknown, the kernel function $k\left(x_{i},x_{j}\right)=Փ\left(x_{i}\right)Փ\left(x_{j}\right)$ was introduced. Kernel functions can take many forms. Commonly used kernel functions include linear kernel functions, polynomial kernel functions, radial basis function kernel, and multilayer perceptron kernel functions. In LDA, for the sample data of a given training set, the original data were mapped onto a straight line. The mapping effects obtained by selecting different straight lines *w* were different. The LDA method needs to find this straight line to ensure that the projection points of similar samples are as close as possible and that the projection points of heterogeneous samples are as distant from each other as possible.

**Figure 5:**
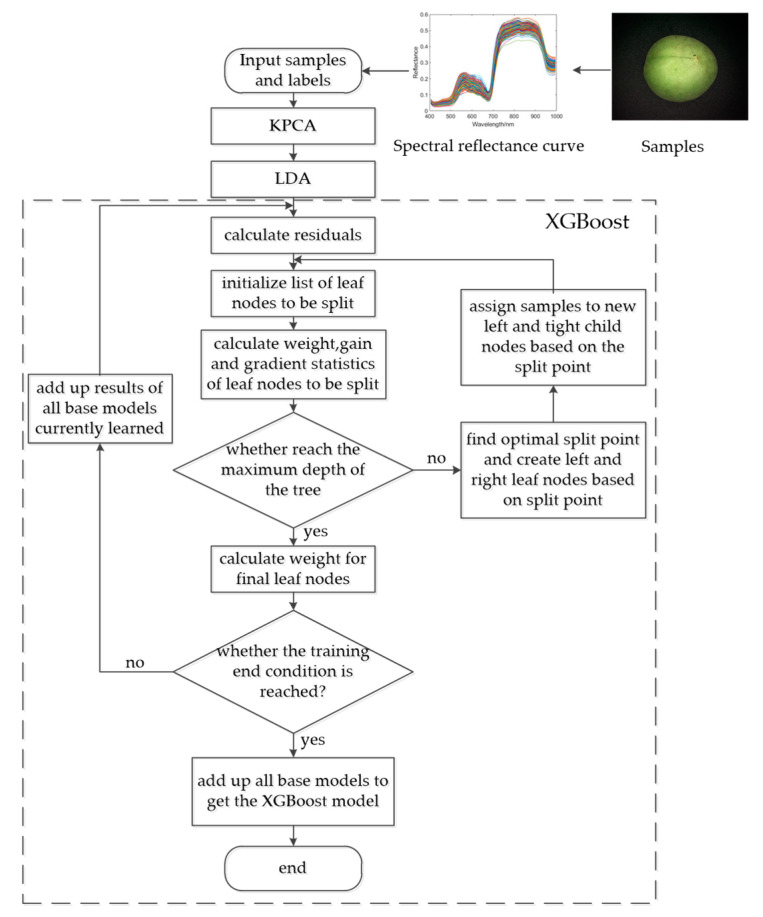
Flow chart of the kernel principle component analysis- linear discriminant analysis- extreme gradient boosting algorithm (KPCA-LDA-XGB) model training.

After performing KPCA and LDA processing on the original spectral data to obtain the feature vector, the XGBoost model was trained via the following process: (1) Input the obtained feature vectors and labels into the model; (2) obtain the residual according to the sum of the predicted value of the currently learned base learner and the true value of the sample, with an initial predicted value of 0; (3) initialize the feature list to be segmented. For potential segmentation points, achieve a change of the objective function before and after segmentation; (4) determine whether the depth of the current base learner reaches the maximum split depth. If it does not reach the maximum depth, find the optimal segmentation point and assign samples to the left and right leaf nodes based on the optimal split point, and then return to step (3). If the maximum split depth is reached, stop splitting, calculate the weight of each leaf node, and complete the establishment of the current base learner; and (5) determine whether the training of the current model reaches the termination condition (i.e., if the number of base learners reaches the set maximum value). If it does not, return to step (2). If it does, combine all the base learners obtained through training to obtain XGBoost. End model training. The XGBoost model training flowchart is shown in Figure 5.

## 4. Results and Discussion

### 4.1. Performance Analysis of Different Kernel Functions

**Figure 6:**
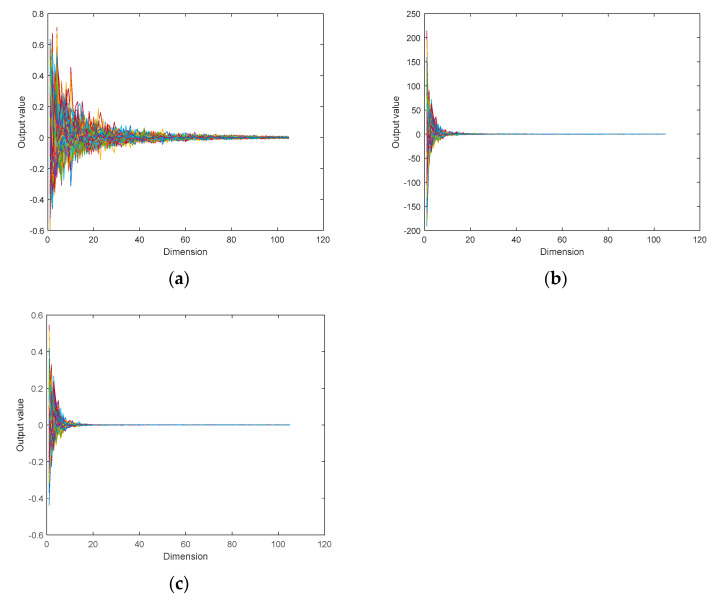
Comparison of the prediction results of the linear kernel function with other kernel functions in the kernel principal component analysis (KPCA) model. (**a**) radial basis function (RBF), (**b**) polynomial (Poly), and (**c**) linear.

In this study, three KPCA kernel functions (linear, radial basis function (RBF), and polynomial (Poly)) were used to reduce the dimensionality of the original data set. The dimensions of the original green plum spectral data were reduced from 119 to 105, and the original data information was retained to the greatest extent. The results are shown in Figure 6, which illustrates the curves of the 366 sets of raw data processed by the three KPCA kernel functions (linear, RBF, and Poly). The horizontal axis represents the dimension of the data, and the vertical axis represents the value of each dimension after processing via the kernel function. Here, the original data after dimensional reduction of the three KPCA kernel functions of linear, RBF, and Poly are similar in their shape and size intervals. The data range processed using the Poly kernel function is larger than that processed using RBF and linear. The data processed using the linear kernel function are also more evenly distributed than those using the RBF kernel function. Therefore, the linear kernel function was used when the original data were processed by KPCA.

**Figure 7:**
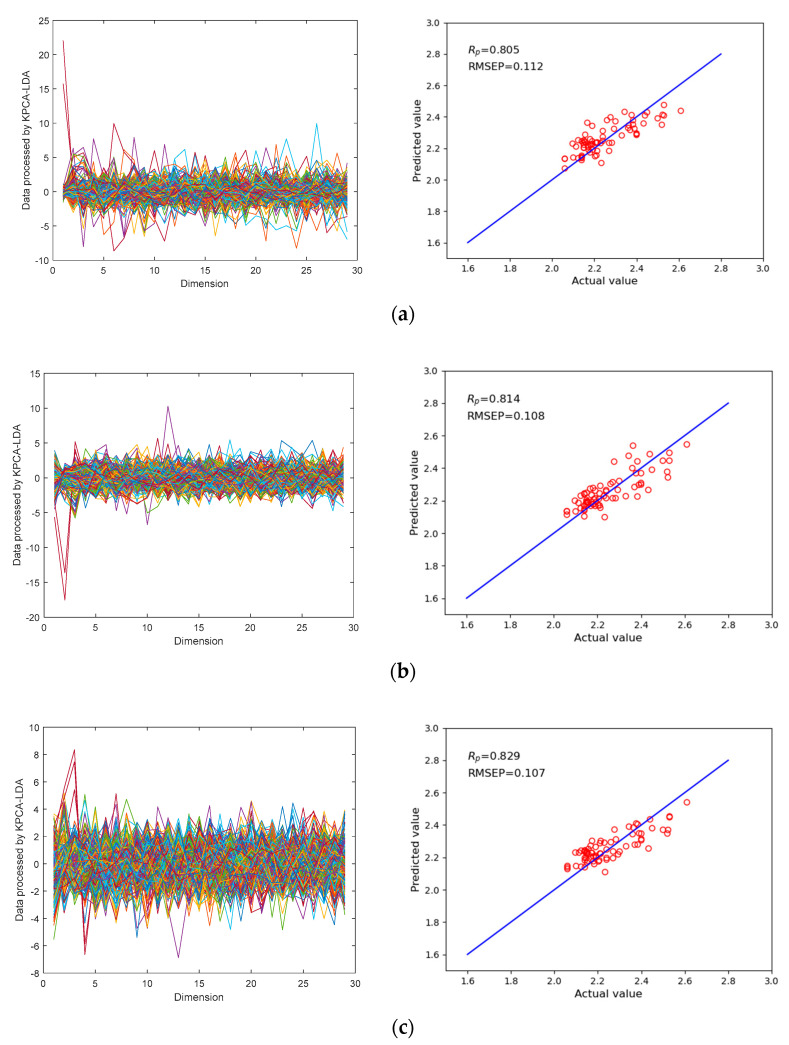
Comparison of the prediction results of the linear kernel function with other kernel functions in the KPCA-LDA-XGB model. (**a**) Prediction results of the KPCA(RBF)-LDA-XGB model. (**b**) Prediction results of the KPCA(Poly)-LDA-XGB model. (**c**) Prediction results of the KPCA(linear)-LDA-XGB model.

To further optimize the performance of the KPCA-LDA-XGB model in predicting the acidity of green plums, LDA was integrated (on the basis of KPCA) to achieve superior supervised feature extraction performance and dimensional reduction of the data. The dimension of the raw spectral data of green plums processed by KPCA was reduced to 29. To evaluate the performance of the current model, the KPCA(RBF)-LDA-XGB, KPCA(Poly)-LDA-XGB, and KPCA(linear)-LDA-XGB models were compared through two indicators: the correlation coefficient in prediction set (***R_P_***) and the root mean squared error in prediction set (RMSEP). Figure 7 shows the prediction effects of different kernel functions in the KPCA-LDA-XGB model, and Table 4 shows the comparison results of the different kernel functions in the KPCA-LDA-XGB model.

The comparison results show that the KPCA(linear)-LDA-XGB model offers better acidity prediction performance for green plum. Compared with KPCA(RBF)-LDA-XGB and KPCA(Poly)-LDA-XGB, the ***R_P_*** of the model were improved by 3.0% and 1.8%, respectively, and the RMSEP was increased by 4.5% and 0.9%, respectively. These results show that the selection of the linear kernel function can determine whether the KPCA-LDA-XGB model will achieve the best performance in predicting the acidity of green plum. Indeed, compared to the other two kernel functions, in KPCA, the linear kernel function was selected. Moreover, the data processed by KPCA-LDA were more uniform in their distribution and had a smaller data distribution range, which gave the model better dimensional reduction capabilities.

### 4.2. Performance Analysis of the KPCA-LDA-XGB Model

The inputs of the KPCA-LDA-XGB model included the hyperspectral characteristic curves of the green plum and the pH value corresponding to 119 dimensions. The number of principal components of the KPCA was set to 105. The chosen kernel function was linear, and the number of principal components of LDA was set to 29. The selected optimization problem algorithm (solver) was the eigen decomposition algorithm (eigen). The actual number of iterations was set to 2000.

The correlation coefficient (R) is a statistical indicator used to reflect the correlation proximity between variables. The value range of R is [–1, 1]. When R=0, there is no linear relationship between variables. When |R|=1, the variables are completely linearly related. When evaluating the prediction ability of the model, if |R|>0.6, then the model can be used for predicting situations with low accuracy requirements. When |R|>0.8, the model has good prediction ability and can complete the required forecasting task.

The root mean squared error (RMSE) is the arithmetic square root of the variance, which can reflect the degree of dispersion of a data set. Therefore, the correlation coefficient *R* and the root mean squared error RMSE were used to evaluate the performance of the KPCA-LDA-XGB model. The correlation coefficient in cross validation set (***R_CV_***) and the root mean squared error in cross validation set (RMSECV) of the KPCA-LDA-XGB model were 0.739 and 0.128, respectively [22]. After training, the ***R_P_*** and RMSEP of the KPCA-LDA-XGB model in the prediction of green plum acidity were 0.829 and 0.107, respectively.

**Figure 8:**
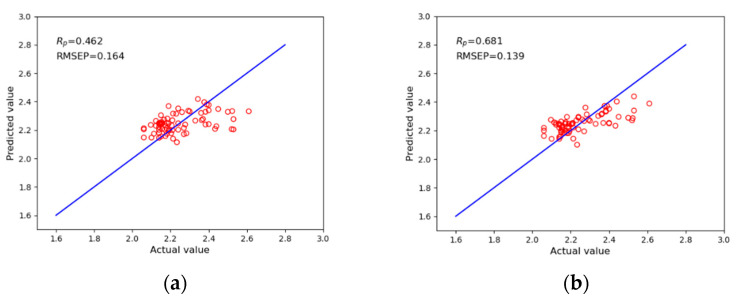

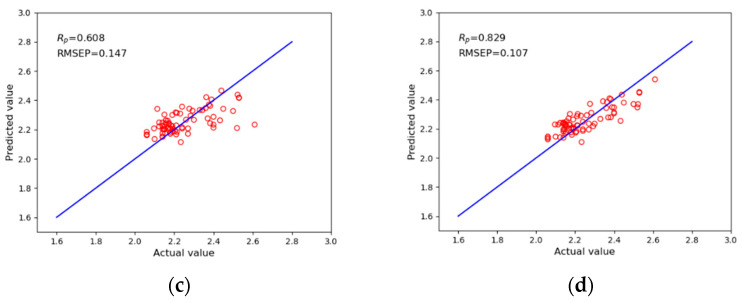
Comparison of the prediction results of the KPCA-LDA-XGB model and the traditional integrated models. (**a**) Prediction results of the XGBoost model (**b**) Prediction results of the KPCA-XGB model. (**c**) Prediction results of the LDA-XGB model. (**d**) Prediction results of the KPCA-LDA-XGB model.

To evaluate the performance of the current model, the KPCA-LDA-XGB model was compared with the three models of XGBoost, KPCA-XGB, and LDA-XGB. Figure 8 shows the prediction effects of the four models, including the XGBoost, KPCA-XGB, LDA-XGB, and KPCA-LDA-XGB models. The ***R_P_*** and RMSEP were used to evaluate the prediction effects of the model. The horizontal axis represents the true value of green plum acidity, and the vertical axis represents the predicted value of green plum acidity. Table 5 shows the comparison results of the KPCA-LDA-XGB model and the three other models.

Compared with XGBoost, KPCA-XGB, and LDA-XGB, the ***R_P_*** of the KPCA-LDA-XGB model increased by 79.4%, 21.7%, and 36.3%, respectively, and the RMSEP decreased by 31.2%, 17.4%, and 12.5%, respectively. These results show that the KPCA-LDA-XGB model offers good performance for predicting green plum acidity. This result occurred primarily because the input of the XGBoost model included the original 119-dimensional spectra data of green plums, while the input of the KPCA-XGB, LDA-XGB, and KPCA-LDA-XGB models was the data after feature extraction and dimensional reduction, including 105, 29, and 29 dimensions, respectively.

**Figure 9:**
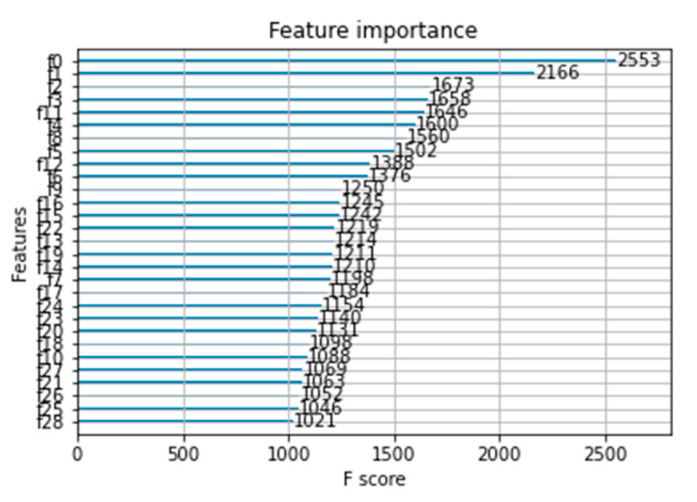
The Selection results of the feature bands of the KPCA-LDA-XGB model.

At the same time, the KPCA-LDA-XGB model achieved an importance ranking of the input 29-dimension data that affect the characteristics of green plum acidity, as shown in Figure 9. The vertical axis uses $f_{0}-f_{28}$ to sequentially represent the 29-dimensional data input of the KPCA-LDA-XGB model. The horizontal axis represents the importance score of each dimensional datum. In the model established to determine the acidity of green plum and extract features based on the XGBoost algorithm, the importance scores were arranged in descending order. $f_{0}$ was the most important parameter affecting the acidity of green plum, while $f_{28}$ was the least important parameter affecting the acidity of green plum.

## 5. Conclusions

Green plum acidity is an important component index considered during the deep processing of green plums. The use of non-destructive testing technology for green plum is of great significance and high practical value for improving the quality of green plum deep processing products, promoting industrial transformations, and upgrading and improving the automation and intelligence levels of green plum production. Using hyperspectral technology, we took the green plum acidity index as the object in this paper to develop a non-destructive testing method for green plum. The experimental analysis proved that the use of visible light/near infrared spectroscopy technology can effectively predict the pH of green plum. Spectral information was collected from 400 to 1000 nm, and the supervised learning model XGBoost was used to predict the acidity of the green plum fruit. The ***R_P_*** and RMSEP of the model were 0.462 and 0.164, respectively. To further improve the prediction accuracy of green plum acidity, the KPCA-LDA-XGB model was proposed based on the XGBoost model. The feature extraction method of the KPCA-LDA-XGB model was improved through the KPCA and LDA methods. The experimental results show that compared with the basic XGBoost model, the KPCA-LDA-XGB model has a 79.4% increase in its ***R_P_*** value and a 31.2% decrease in its RMSEP. Compared with KPCA-XGB and LDA-XGB, which use KPCA or LDA alone for feature extraction and data dimensional reduction, the ***R_P_*** of the KPCA-LDA-XGB model increased by 21.7% and 36.3%, respectively, and the RMSEP were reduced by 17.4% and 12.5%, respectively. Ultimately, the proposed KPCA-LDA-XGB model offers good prediction performance for the acidity of green plums. Since the amount of collected green plum data was not very large, this sample set could be increased in future research to reduce randomness. More machine learning models could also be used to further improve the accuracy of the model.

## Figures and Tables

**Table 1:** Parameters of Gaiafield-V10E-AZ4 hyperspectral camera.

Specifications	Parameters
Spectral range	400–1000 nm
Spectral resolution	2.8 nm
Data output	16 bits
Data interface	USB3.0/CameraLink

**Table 2:** Acidity distributions of green plum.

Sample Set	*n*	Max	Min	Mean	SD
Calibration set	274	2.74	2.03	2.26	0.1399
Prediction set	92	2.71	2.04	2.27	0.1327

Note: SD means standard deviation.

**Table 3:** Software and hardware environment configuration.

Name	Parameter
System	Windows 10 × 64
CPU	Inter I9 9900K@3.60 GHz
GPU	Nvidia GeForce RTX 2080 Ti(11G)
Environment configuration	PyCharm + Tensorflow 2.1.0 + Python 3.7.7 Cuda 10.0 + cudnn 7.6.5 + XGBoost 1.1.1
RAM	64 GB

**Table 4:** Comparison of the prediction and cross validation performance of the linear kernel function with the other kernel functions in the KPCA-LDA-XGB model.

Model	*R_P_*	RMSEP	*R_CV_*	RMSECV
KPCA(RBF)-LDA-XGB	0.805	0.112	0.744	0.128
KPCA(Poly)-LDA-XGB	0.814	0.108	0.753	0.126
KPCA(linear)-LDA-XGB	0.829	0.107	0.739	0.128

**Table 5:** Comparison of the prediction and cross validation performance of the KPCA-LDA-XGB model and the traditional integrated models.

Model	*R_P_*	RMSEP	*R_CV_*	RMSECV
XGBoost	0.462	0.164	0.214	0.186
KPCA-XGB	0.681	0.139	0.589	0.155
LDA-XGB	0.608	0.147	0.655	0.144
KPCA-LDA-XGB	0.829	0.107	0.739	0.128

## Data Availability

The data are not publicly available due to the company requirements.
